# Supplementary material for: Virtual Reality-Based Cognitive Stimulation on People with Mild to Moderate Dementia due to Alzheimer’s Disease: A Pilot Randomized Controlled Trial
Source: Int J Environ Res Public Health. 2021 May 16;18(10):5290. doi: 10.3390/ijerph18105290 (PMC8156930; doi:10.3390/ijerph18105290)
Supplement: Supplementary file 1 [file ijerph-18-05290-s001.zip › ijerph-1197969.pdf]

International Journal of
Environmental Research and Public Health

MDPI

Table 1. Structure of the intervention protocol.

Session #	Structure	Proposed cognitive domains
Session 1	Training	N/A
Session 2	T1 and T2	Prospective memory (T1), planning and attention (T2)
Session 3	T3 and T4 (L1)	Planning (T3), visual memory (T4)
Session 4	T4 (L2) T5 (L1)	Visual memory (T4), executive functions, memory/attention (T5)
Session 5	T5 (L2)	Executive functions, memory and attention
Session 6	T6	Declarative memory
Session 7	T7 (L1)	Executive functions, working memory
Session 8	T7 (L2)	Executive functions, working memory
Session 9	T8 (L1)	Executive functions, working memory
Session 10	T9 (L1)	Attention, working memory
Session 11	T9 (L2)	Attention, working memory
Session 12	T9 (L3)	Attention, working memory

Note: T1 Morning hygiene, T2 Shoe closet test, T3 Wardrobe test, T4 Memory test, T5 Virtual kitchen, T6 TV news, T7 Grocery store, T8 Pharmacy, and T9 Art gallery test.

Annex 1

CONSENTIMENTO INFORMADO, LIVRE E ESCLARECIDO PARA PARTICIPAÇÃO EM INVESTIGAÇÃO de acordo com a Declaração de Helsínquia e a Convenção de Oviedo[1]

Por favor, leia com atenção a seguinte informação. Se achar que algo está incorreto ou que não está claro, não hesite em solicitar mais informações. Se concorda com a proposta que lhe foi feita, queira por favor assinar este documento.

Título do estudo: Estudo sobre os benefícios da Estimulação Cognitiva com recurso à Realidade Virtual em indivíduos com Demência leve ou moderada.

Enquadramento: No âmbito de um projeto de investigação conjunto entre a Escola de Psicologia e Ciências da Vida da Universidade Lusófona de Humanidades e Tecnologias e a Santa Casa da Misericórdia da Amadora, sob coordenação de Prof. Dr. Pedro Gamito e Prof. Dr. Jorge Oliveira, pretende-se estudar se existem melhorias a nível cognitivo com o treino em tarefas de realidade virtual em pessoas com diagnóstico de demência.

Explicação do estudo: Para o desenvolvimento da investigação, que terá a duração de 4 semanas (cuja sessões, serão 3 vezes por semana, com duração de 45 minutos), será necessário constituir dois grupos de utentes, com demência (leve ou moderada) que irão realizar exercícios em realidade virtual.
Os participantes deverão preencher o protocolo de avaliação em dois momentos diferentes no estudo. Este protocolo é constituído por questionários e provas de papel-e-lápis que duram cerca de 30 minutos a preencher. Estes testes não apresentam qualquer risco e não implicam qualquer tipo de desconforto. No entanto, pode ser sentido algum grau de cansaço ao longo deste tempo, pelo que estão previstas interrupções se assim for desejado.

Condições e financiamento: Este projeto não envolve contrapartidas financeiras. A participação nesta investigação é assim voluntária e se o participante decidir não participar ou quiser desistir de continuar em qualquer momento, tem absoluta liberdade de fazê-lo sem que isso afete o tratamento normal ao qual tem direito. Será assegurado o livre acesso a todas as informações e esclarecimentos adicionais sobre o estudo, antes, durante e depois da participação. No final da investigação, será proporcionado um resumo dos resultados em formato que seja compreensível para os participantes.

Confidencialidade e anonimato: Assegura-se que será mantido o anonimato e a confidencialidade dos dados pessoais, pois consagra-se como obrigação e dever o sigilo profissional bem como o uso exclusivo dos dados recolhidos para o presente estudo. Serão omitidas todas as informações que permitam uma identificação pessoal. Todos os contatos estabelecidos com os participantes serão feitos em ambiente de privacidade.

Benefícios da participação: Espera-se que ao nível dos benefícios se encontre melhorias após esta intervenção dirigida à promoção da função cognitiva em pessoas com demência. Além deste benefício, espera-se igualmente contribuir para a compreensão do fenómeno estudado e para a produção de conhecimento científico sobre esta condição e formas de minimizar as suas consequências, bem como, de promover a qualidade de vida em pessoas com demência.

Agradeço a sua colaboração.

Pedro Gamito (Investigador responsável – pedro.gamito@ulusofona.pt)

Jorge Oliveira (Investigador – jorge.oliveira@ulusofona.pt)

[1] http://dre.pt/pdf1sdip/2001/01/002A00/00140036.pdf

Annex 2

CONSENTIMENTO INFORMADO DO PROFISSIONAL DE SAÚDE RESPONSÁVEL

Pelo presente instrumento, declaro que fui suficientemente esclarecido (a) pelo técnico sobre os procedimentos (método da investigação e instrumentos de recolha de dados) a que vai ser submetido (a) *(nome do participante),* do qual sou profissional de saúde com da área da Neuropsicologia, bem como dos objetivos da investigação que visa comprovar os benéficos de tarefas de estimulação cognitiva em realidade virtual para pessoas com demência leve ou moderada.

Declaro, também, que fui informado(a) que a participação será voluntaria e que os dados são exclusivamente para dados de investigação e, que todas as informações recolhidas são absolutamente confidenciais e estarão abrangidas pelo segredo profissional.

Foi-me transmitido que os procedimentos seguem as normas éticas, não oferecendo riscos de qualquer natureza, uma vez que os instrumentos de recolha de dados não implicam desconforto ou dor nem afetam desfavoravelmente a saúde do(a) participante bem como serei informado(a) imediatamente sobre possíveis alterações/problemas que porventura possam surgir.

Pelo presente manifesto expressamente minha concordância e meu consentimento para realização da investigação acima descrita, tendo em conta, também, que o mesmo é do assentimento do participante e do seu representante legal.

Nome: … … … … … … … …... … … … …... … … … … … … … … … … … …

Assinatura: … … … … … … … …... … … … … … … … … …

Data: …… /…… /………..

ESTE DOCUMENTO É COMPOSTO DE DUAS PÁGINAS E FEITO EM DUPLICADO:
UMA VIA PARA A INVESTIGADORA, OUTRA PARA A PESSOA QUE CONSENTE

Annex 3

CONSENTIMENTO INFORMADO DO CUIDADOR

Pelo presente instrumento, declaro que fui suficientemente esclarecido (a) pelo técnico sobre os procedimentos (método da investigação e instrumentos de recolha de dados) a que vai ser submetido (a) *(nome do participante),* do qual sou cuidador informal, como dos objetivos da investigação que visa comprovar os benéficos de tarefas de estimulação cognitiva em realidade virtual para pessoas com demência leve ou moderada.

Declaro, também, que fui informado(a) que a participação será voluntaria e que os dados são exclusivamente para dados de investigação e, que todas as informações recolhidas são absolutamente confidenciais e estarão abrangidas pelo segredo profissional.

Foi-me transmitido que os procedimentos seguem as normas éticas, não oferecendo riscos de qualquer natureza, uma vez que os instrumentos de recolha de dados não implicam desconforto ou dor nem afetam desfavoravelmente a saúde do(a) participante bem como serei informado(a) imediatamente sobre possíveis alterações/problemas que porventura possam surgir.

Pelo presente manifesto expressamente minha concordância e meu consentimento para realização da investigação acima descrita, tendo em conta, também, que o mesmo é do assentimento do participante e do seu representante legal.

Nome: … … … … … … … …... … … … …... … … … … … … … … … … … … …

Assinatura: … … … … … … … …... … … … … … … … … …

Data: …… /…… /………..

ESTE DOCUMENTO É COMPOSTO DE DUAS PÁGINAS E FEITO EM DUPLICADO:
UMA VIA PARA A INVESTIGADORA, OUTRA PARA A PESSOA QUE CONSENTE
